# The esoteric roles of Bcl-2 family proteins in glucose homeostasis and cell survival

**DOI:** 10.1038/cddis.2015.320

**Published:** 2015-11-05

**Authors:** S Pfeiffer, J H M Prehn

**Affiliations:** 1 Department of Physiology and Medical Physics, Centre for Systems Medicine, Royal College of Surgeons in Ireland, 123 St. Stephen's Green, Dublin 2, Ireland

The prevalence and the incidence of type 2 diabetes mellitus (T2DM) are increasing and now attaining epidemic proportions worldwide. Apoptotic cell death is a hallmark of pancreatic beta-cell loss not only in type 1 diabetes mellitus (T1DM) but also in all forms of diabetes mellitus, contributing to the development of clinically overt disease.^1, 2^ Although activation of pro-apoptotic Bcl-2 protein family members Bax, Bim, Puma and Bad has been identified to initiate beta-cell apoptosis in *in vitro* and *in vivo*,^3, 4, 5^ recent years have seen the gradual elucidation of additional roles played by Bcl-2 family proteins in the control of beta-cell energetics and glucose-stimulated insulin secretion, indicative of a role for Bcl-2 proteins in beta-cell physiology.^6, 7, 8^

In a recent study published in *Cell Death Discovery*,^9^ we not only demonstrate a role for pro-apoptotic Bcl-2 homology domain-3-only protein Bmf (Bcl-2-modifying factor) as a mediator of beta-cell apoptosis in a mouse model of a monogenic form of diabetes, HNF1*α*-MODY, but also reveal an extracurricular role for Bmf in beta-cell insulin secretion unrelated to cell death initiation *in vivo* (Figure 1). Hepatocyte nuclear factor-1A maturity-onset diabetes of the young (HNF1*α*-MODY) is the most common monogenic form of diabetes and is characterized by a primary *β*-cell failure without features of insulin resistance. The study follows on from previous work demonstrating an AMP-activated protein kinase (AMPK)-dependent induction of Bmf expression during energetic stress in insulin-secreting cells and in the islets of transgenic mice expressing a dominant-negative (DN) mutant of the transcription factor HNF-1*α* in beta cells, identifying Bmf as a primary mediator of beta-cell apoptosis in HNF1*α*-MODY.^10^ Through the introduction of *bmf*-deficiency in a transgenic HNF1*α*-MODY mouse model, we report an attenuation in beta-cell loss and preserved insulin content in *bmf*-deficient diabetic mice. Intriguingly, *bmf*-deficiency was found to exacerbate rather than reduce hyperglycemia and ultimately resulted in a decreased glucose-stimulated insulin response, implicating a role for Bmf in glucose homeostasis regulation in addition to effects on beta-cell survival. Indeed, a decreased glucose-stimulated insulin response was also observed in *bmf-*deficient non-MODY control mice.

This work expands on current re-thinking of the roles of Bcl-2 family proteins and their functional involvement in physiological pathways other than cell death and survival.^11^ Regulation of cellular metabolic functions in addition to regulation of apoptosis in beta cells has previously been observed in the case of the BH3-only protein Bad, which regulates mitochondrial associated glucokinase activity and mitochondrial respiration in beta cells.^7^ Anti-apoptotic Bcl-2 family proteins Bcl-2 and Bcl-xl have also been shown to impact on mitochondrial nutrient metabolism and insulin secretion signaling by dampening the beta-cell response to glucose.^6^ This was observed in the absence of Bax and Bak expression (indicating these were independent of apoptosis signaling), and was attributed to effects of Bcl-xL and Bcl-2 on ER and Ca^2+^ homeostasis. As both Bcl-2 and Bcl-xL regulate mitochondrial fusion, effects of Bcl-2 and Bcl-xL on mitochondrial morphology, bioenergetics or quality control could have likewise contributed. Bmf is capable of binding Bcl-2 and Bcl-xL.^12^ In accordance with the observations of the Johnson group, we demonstrate an impairment of glucose-stimulated insulin secretion in *bmf-*deficient mice, and a resultant aggravated hyperglycemia that was evident not only in HNF1*α*-MODY mice but also in the control mice generated through a rigorous backcrossing strategy. Although the rat insulin promoter (RIP) system targeted DNHNF-1*α* expression to beta cells (and potentially other areas of the brain in which HNF1*α* is not highly expressed), it should be noted that the *bmf* knockout in this model resulted in gene deficiency in all tissues potentially impacting on glucoregulation.

Interestingly, female mice in this model do not manifest the same overt diabetic phenotype as male mice. The sexual dimorphism observed in this model has been paralleled in many other models of diabetes.^13^ Activation of estrogen receptors in rodent models of T1DM and T2DM has been shown to promote *β*-cell survival, indicating that the hormonal profile of female animals appears to confer protection from the development of overt diabetes.^13, 14^ Interestingly, female mice, that is, MODY mice were protected from beta-cell loss, but were unprotected from loss of glucose-stimulated insulin secretion upon deletion of *bmf.*

Taken together, the results of this study conclude that deletion of *bmf* results in a rescue of the progressive beta-cell death in a mouse model of monogenic diabetes, but surprisingly also plays a role in glucose homeostasis with an observable insulin secretory defect *in vivo*. This work provides an insight into the ‘day-night' dual role played by Bmf and other BH3-only proteins in the control of *in vivo* glucose homeostasis.

## Figures and Tables

**Figure 1:**
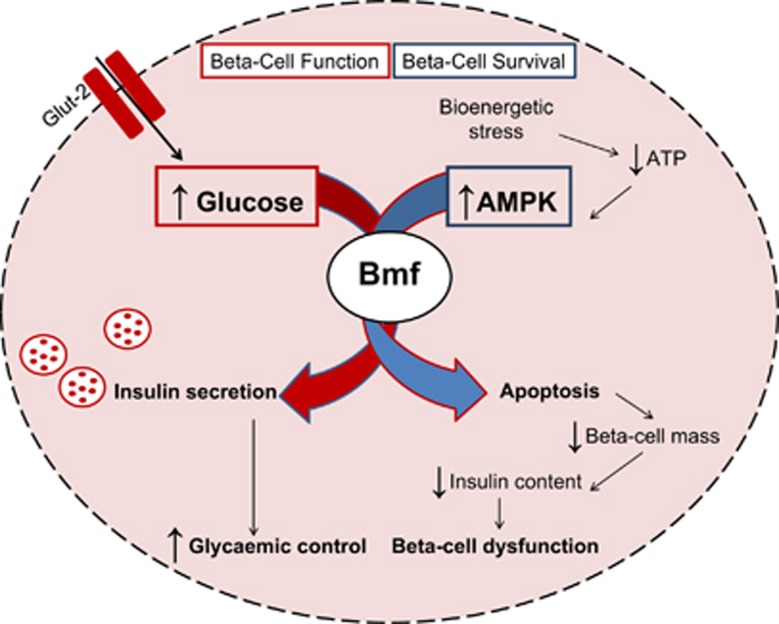
The dual roles for pro-apoptotic Bcl-2 homology domain-3-only protein Bmf (Bcl-2-modifying factor) as a mediator of beta-cell apoptosis and beta-cell insulin secretion *in vivo*: Bmf is required for glucose-stimulated insulin secretion *in vivo*, thereby maintaining glycemic control (red arrow). Depletion of ATP and prolonged activation of AMPK, however, also triggers Bmf-mediated apoptosis of beta cells, resulting in decreased beta-cell mass and corresponding reduction in islet insulin content (blue arrow)
